# Effectiveness and Safety of Pharmacopuncture on Inpatients with Tension Headache Caused by Traffic Accidents: A Pragmatic Randomized Controlled Trial

**DOI:** 10.3390/jcm13154457

**Published:** 2024-07-30

**Authors:** Ja-Yean Son, Kangmoo Goo, Na-young Kim, Seok-Gyu Yang, Dong Hwan Lee, Yu-Ra Im, Oh Bin Kwon, Hyun-Woo Cho, Sang Don Kim, Doori Kim, In-Hyuk Ha

**Affiliations:** 1 Department of Korean Medicine Rehabilitation, Haeundae Jaseng Hospital of Korean Medicine, 793 Haeun-daero, Haeundae-gu, Busan 48102, Republic of Koreasilica715@naver.com (S.-G.Y.); kamui0328@jaseng.org (H.-W.C.); donmuta@naver.com (S.D.K.); 2 Department of Korean Medicine Neuropsychiatry, Haeundae Jaseng Hospital of Korean Medicine, 793 Haeun-daero, Haeundae-gu, Busan 48102, Republic of Korea; 3 Department of Korean Internal Medicine, Haeundae Jaseng Hospital of Korean Medicine, 793 Haeun-daero, Haeundae-gu, Busan 48102, Republic of Korea; roll0123@naver.com; 4 Department of Korean Acupuncture & Moxibustion Medicine, Haeundae Jaseng Hospital of Korean Medicine, 793 Haeun-daero, Haeundae-gu, Busan 48102, Republic of Korea; asde112_@naver.com (D.H.L.); ohbin5325@naver.com (O.B.K.); 5 Department of Korean Medicine Ophthalmology & Otolaryngology & Dermatology, Haeundae Jaseng Hospital of Korean Medicine, 793 Haeun-daero, Haeundae-gu, Busan 48102, Republic of Korea; yura5795@naver.com; 6 Clinical Research Center, Jaseng Hospital of Korean Medicine, 536, Gangnam-daero, Gangnam-gu, Seoul 06110, Republic of Korea; 7 Jaseng Spine and Joint Research Institute, Jaseng Medical Foundation, 2F 540 Gangnam-daero, Gangnam-gu, Seoul 06110, Republic of Korea

**Keywords:** pharmacopuncture, tension headache, traffic accident

## Abstract

**Background:** This study investigated the effectiveness and safety of pharmacopuncture for pain relief and functional improvement in patients with traffic accident (TA)-induced acute tension headaches. **Methods:** The study employed a parallel, single-centered, pragmatic, randomized controlled trial design. Eighty patients complaining of acute tension headaches were randomized into the integrative Korean medicine treatment (IKM treatment) group and the pharmacopuncture group on suboccipital muscles (suboccipital muscles pharmacopuncture + IKM treatment), with 40 participants assigned to each group. The patients in the pharmacopuncture group underwent pharmacopuncture as an add-on therapy, consisting of three sessions. Both groups were reassessed 2 months post-intervention. To assess the outcomes, the Numeric Rating Scale (NRS) for Headache, NRS for Neck Pain, Headache Disability Index, Headache Impact Test-6, EuroQol 5-Dimension, and Patient Global Impression of Change were used. **Results:** The improvement in the outcomes of the pharmacopuncture group was significantly greater than that of the comparison group on day 4 of hospitalization in terms of pain (difference in NRS of headache −2.59, 95% CI −3.06 to −2.12; NRS of Neck pain −1.05, 95% CI −1.50 to −0.59) and function (difference in HDI −24.78, 95% CI, −31.79 to −17.76; HIT-6 −6.13, 95% CI, −9.47 to −2.78). Additionally, in 2 months of follow-up, the recovery rate of headache was significantly higher in the pharmacopuncture group than in the comparison group. **Conclusions:** The pharmacopuncture group demonstrated superior outcomes in symptom improvement than the comparison group did, providing insights into novel and useful applications of pharmacopuncture in the clinical practice of Korean medicine.

## 1. Introduction

Whiplash injury refers to damage to the bone and tissue caused by the mechanism by which energy generated from sudden, forceful acceleration-deceleration is transferred to the bone or soft tissues in the neck [1,2]. Whiplash-associated disorders (WADs) are a collection of symptoms, including neck pain, headache, dizziness, sleep disturbance, vertigo, fatigue, and insomnia, which are developed after sustaining whiplash injury [2]. Among the wide range of symptoms of WADs, as described above, headache is the second most common after neck pain [3,4]. A previous study reported that more than about 60% of patients complained of a headache within 7 days after a whiplash injury, and approximately 38% of patients continued to report headaches one year after the whiplash injury occurred [3]. Another study reported that 50% of patients continued to complain of whiplash-associated disorders (WAD) one year after the whiplash injury occurred, and nearly 40% of patients continued to report headaches even after 5 years [5].

Since the symptoms of WADs vary depending on the progression of the condition (acute or chronic stage), the selection of optimal treatment methods for the symptoms is imperative [6,7]. Tension-type headaches (TTH) refer to mild to moderate intensity headaches with bilateral pressure or squeezing sensation, and the most significant clinical feature is sustained tenderness of the cranio-cervical muscles. Trigger points (TrPs) and muscle tenderness in TTH have been recognized as associated with onset factors. TrPs in the neck muscles could influence the muscle imbalance of the major muscles of the head [8]. On the other hand, the suboccipital muscles have been reported to maintain head stability while allowing delicate comparison of the movement of the atlanto-occipital and atlanto-axial joints with a weak, sustained force [9]. Previous studies have reported an association between suboccipital muscles and headache and neck pain [9,10,11,12]. The development of trigger points in the suboccipital muscles can lead to muscle imbalance and instability in the head and neck, along with referred pain patterns to the head and cervical region [9,10,11,12,13].

Treatment options for TTH include pharmacotherapy, physical therapy, psychological therapy, and exercise [13]. Previous studies reported that suboccipital muscle inhibition or soft tissue manipulation significantly reduced neck and head pain as well as disability in patients with TTH [14,15]. According to the clinical practice guidelines of Korean Medicine, acupuncture, electroacupuncture, pharmacopuncture, motion-style acupuncture treatment, Chuna therapy, moxibustion, cupping, and herbal medicine therapy are recommended as treatment options for WADs because of traffic injuries [6].

On the other hand, pharmacopuncture is one of the treatment methods of traditional Korean medicine that combines pharmacology and acupuncture, which benefits from the concurrent effects of needling and pharmacologic actions by injecting a needle loaded with the extraction of herbal medicine prepared in a specific prescribed method into positive reaction points [16]. Pharmacopuncture is widely used for many different conditions, including musculoskeletal diseases [6,17,18,19]. In Korea, a retrospective study of medical charts for 342 patients with traffic accident (TA) injuries who visited a Korean medicine clinic reported that 298 patients, accounting for 99.42% of the total, received pharmacopuncture. [4] Furthermore, another previous study investigated 845 TA patients with TA-associated musculoskeletal symptoms who received Korean medicine treatments; 668 patients received pharmacopuncture, and 533 (79.7%) of them considered pharmacopuncture as the most satisfactory treatment [20].

Despite the high frequency of use and high level of treatment satisfaction with pharmacopuncture in clinical practice in Korea, there is still a paucity of evidence regarding the effectiveness of pharmacopuncture for WADs. Most existing literature has mainly investigated lumbar or cervical spinal diseases, such as neck and lower back pain [6].

Therefore, this study was conducted to examine the effectiveness and safety of pharmacopuncture on suboccipital muscles in patients with TA-induced an acute tension headache. We hypothesized that combined pharmacopuncture and IKM treatment would be more effective than IKM treatment alone.

## 2. Methods

### 2.1. Study Protocol

All participants were properly informed and given a thorough explanation by the researchers before participating in the study. They then signed a consent form. This study was approved by the Investigational Review Board of the Jaseng Hospital of Korean Medicine (Approval No.: 2022-08-049), and the study protocol is registered at ClinicalTrial.gov (NCT05549765).

### 2.2. Study Design

This study was designed as a parallel, single-centered, pragmatic, randomized controlled trial. The study participants were recruited from among patients hospitalized at the Haeundae Jaseng Hospital of Korean Medicine following traffic accidents from September 2022 to August 2023.

Eighty patients were randomized at a ratio of 1:1, with 40 patients assigned to the suboccipital muscle pharmacopuncture group (pharmacopuncture group) and the IKM treatment group (comparison group). Patients in both groups underwent acupuncture, pharmacopuncture, herbal medicine, and Chuna therapy during their hospital stay. In the pharmacopuncture group, pharmacopuncture of the suboccipital muscles was administered as an add-on therapy once daily from hospitalization day 2 to day 4, making three sessions in total. However, pharmacopuncture in the comparison group was administered in areas other than the suboccipital region. Follow-up assessments were conducted on the day of discharge and 2 months after enrollment (Supplementary Table S1).

### 2.3. Participants

#### 2.3.1. Inclusion Criteria

(1) Male and female patients ages 19–69 years
(2) Patients with acute tension headache that occurred within 7 days after a TA
(3) Inpatients for treatment of TA injuries
(4) Patients with Numeric Rating Scale (NRS) score ≥ 5 for headache
(5) Patients who provide voluntary consent to participate in the trial and return the signed informed consent form

#### 2.3.2. Exclusion Criteria

(1) Diagnosis of a specific critical condition that may cause headache, such as malignancy, cerebral hemorrhage, epidural or subdural hematoma, subarachnoid hemorrhage, and so on
(2) Progressive neurological deficits or severe neurological symptoms
(3) The cause of pain is because of a problem of the nervous system and not the suboccipital muscles, trigeminal neuralgia, glossopharyngeal neuralgia, postherpetic neuralgia, and so on
(4) Patients who have had head surgery or procedures within the last 3 weeks
(5) Other chronic conditions that may interfere with the interpretation of the therapeutic effects or results, including cardiovascular disease, kidney disease, diabetic neuropathy, dementia, epilepsy, and so on
(6) Currently taking steroids, immunosuppressants, medication for mental illness, or other drugs that may affect the results of the study
(7) When administration of pharmacopuncture is inadequate or unsafe: patients with hemorrhagic diseases, those on anticoagulants, those with severe diabetes with a risk of infection, and those with severe cardiovascular disease
(8) Pregnant or planning to become pregnant
(9) Participating in clinical trials other than observational studies without therapeutic intervention
(10) Patients with difficulties in signing the informed consent form
(11) Other patients whose participation in the trial is deemed problematic by the researcher

### 2.4. Randomization and Blinding

Randomization was performed by a screening researcher. The eligibility of the participants who voluntarily signed the informed consent form was determined by evaluation against the inclusion/exclusion criteria. When they were determined to be eligible for the study, a random number table generated by a statistician was used for randomized allocation to the pharmacopuncture group and the comparison group, thus assigning 40 participants to each group (at a ratio of 1:1). Block randomization was used for random sequences. The size of one block was randomly set to 2, 4, or 6. The generated randomization results were sealed in opaque envelopes and stored in a double-locked cabinet by a third party unrelated to the study. A screening researcher opened a sealed randomization envelope to allocate the groups.

Blinding the participants and physicians was not possible because of the nature of the study design, so single blinding was performed for the assessor only. As for the assessor, a study nurse or resident of the relevant specialty who did not participate in the intervention procedures and was blinded to the group assignment performed assessments after the intervention in a separate area.

### 2.5. Interventions

IKM treatment and pharmacopuncture on the suboccipital muscles were administered by Korean medicine doctors (KMDs) with no less than 3 years of clinical experience who acquired their KMD license upon completing the 6-year course at the College of Korean Medicine. All KMDs who performed the interventions completed systematic, standardized training in Chuna therapy and pharmacopuncture. The treatment modalities included in the IKM treatment were determined based on the “Clinical Practice Guideline of Korean Medicine for Tension-Type Headache” and the “Clinical Practice Guideline of Korean Medicine for Traffic Injuries”.

#### 2.5.1. Treatment Course

The patient’s treatments were divided into morning and afternoon sessions, with therapy conducted twice daily. The morning session consisted of Chuna therapy, pharmacopuncture, and acupuncture treatment. After 10–15 min of Chuna therapy, there was a 10-min rest before concurrent pharmacopuncture and acupuncture treatment. In this instance, pharmacopuncture was administered to areas apart from the occipital region or the suboccipital muscles. The morning session was conducted similarly for the pharmacopuncture and comparison groups. The comparison group received only acupuncture treatment during the afternoon session, excluding pharmacopuncture and Chuna therapy. In the pharmacopuncture group, from the 2nd to the 4th day of hospitalization, concurrent pharmacopuncture on the suboccipital muscles and acupuncture treatment were administered. Afterwards, the treatment proceeded similarly to the comparison group.

#### 2.5.2. Comparison Group: IKM Treatment Group

**Figure 1:**
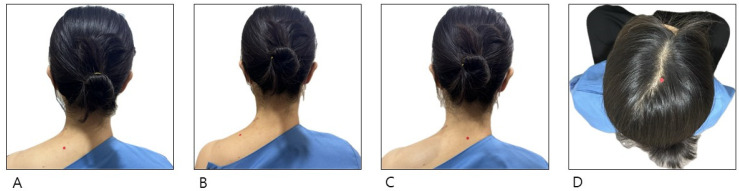
Location of SI14 (肩外兪) GB21 (肩井), GV14 (大椎), and GV20 (百會). (**A**): SI14 (肩外兪), l located laterally 3 cun (寸) from the posterior median line, at the level of the lower border of the spinous process of the 1st thoracic vertebra; (**B**): GB21 (肩井), located at the midpoint of the line connecting the spinous process of the 7th cervical vertebra and the lateral end of the acromion; (**C**): GV14 (大椎), located on the posterior median line, below the spinous process of the 7th cervical vertebra in a depression; (**D**): GV20 (百會), located 5 cun (寸) above the anterior hairline on the anterior median line of the head.

The comparison group underwent integrative Korean medicine treatment (IKM treatment), which consisted of acupuncture, pharmacopuncture, Chuna therapy, and herbal medicine therapy during hospitalization. Sterilized, disposable stainless-steel needles (0.30 × 40 mm, Dongbang Acupuncture, Korea) were used for acupuncture. The essential acupuncture points used were bilateral SI14, GB21, GV14, and (百會) (Figure 1). Additionally, Ashi points and cervical Hyeopcheok acupoints were utilized depending on the need. The points SI14 and GB21 were needled perpendicularly, while GV14 and GV20 were needled obliquely. The physicians selected the method and depth of needling for the remaining acupuncture points based on the points’ anatomical characteristics and the patient’s physique. After needling, *de qi* [21] (participant’s subjective sensations and objective body responses) was elicited through twirling manipulation, followed by a 15-min retention of the needles. Acupuncture treatment was administered twice daily.

Chuna therapy is a manipulation technique of Korean medicine in which a KMD uses their hand, other parts of the body, or assistive devices, such as a Chuna table, to apply effective stimulation to the physical structure of a patient for treatment of structural or functional problems. The techniques used in Chuna therapy include joint mobilization, distraction, myofascial technique, and correction/adjustment [22,23]. During the hospitalization period, Chuna therapy was administered once daily for 10–15 min.

For herbal medicine, a decoction of herbal extracts prepared by formulation of medicinal herbs with the efficacy of promoting blood circulation (活血), regulation of qi (理氣), replenishing blood supply (補血), pain relief (鎭痛), mind-calming/soothing (安神), and removing blood stasis (去瘀) were packed into 75 mL pouches. The patients took the prepared herbal medicine twice daily in the morning and afternoon, 30 min after each meal.

#### 2.5.3. Pharmacopuncture Group: A Group with an Add-On Therapy of Pharmacopuncture on Suboccipital Muscles (Suboccipital Muscles Pharmacopuncture + IKM Treatment)

All participants in the pharmacopuncture group underwent the same IKM treatment as the comparison group during the hospitalization period. Additionally, these patients underwent pharmacopuncture on the suboccipital muscles once daily on days 2, 3, and 4 of hospitalization for three sessions. For the administration of pharmacopuncture, sterilized disposable needles (26 G, 13 mm, Sungshim Medical, Bucheon-si, Republic of Korea) and syringes (Luer Slip, Luer Lock, 3 mL/cc, Sungshim Medical) were used. The KMD administered pharmacopuncture on trigger points (TrPs) of the suboccipital muscles and tender points where palpation felt tenderness. One of the four types of solution, Shinbaro (JS3-SBO, hGMP *Paeoniae Radix alba*), hGMP *Notopterygii Radix et Rhizome,* hGMP *Angelicae pubescentis Radix*, hGMP *Eucommiae Cortex Preparata cum Sal*, hGMP *Cyathulae Radix*, hGMP *Cibotii Rhizoma*, hGMP *Saposhnikoviae Radix*, hGMP *Acanthopanacis Cortex*, hGMP *Scolopendra subspinipes*), Myofascial-release (JS4-M, hGMP *Paeoniae Radix alba*, hGMP *Glycyrrhizae Radix et Rhizoma*), Jungseongeohyeol (A2-JS, hGMP *Sappan Lignum*, hGMP *Salviae Miltiorrhizae Radix*, hGMP *Paeoniae Lactiflorae*, hGMP *Persicae Semen*, hGMP *Sulfur*, hGMP *Commiphora molmol Engler*, hGMP *Corydalis Rhizoma*, hGMP *Gardeniae Fructus*), or Hwangryunhaedoktang (A1-HR, hGMP *Coptidis Rhizoma*, hGMP *Scutellariae Radix*, hGMP *Phellodendri Cortex*, hGMP *Gardeniae Fructus*) was selected and administered. A volume of 0.2 to 0.5 cc of solution was injected per acupoint. The physician’s clinical judgment determined the type of solution, dosage, and depth of pharmacopuncture, considering the anatomical characteristics of the acupoints and the patient’s physique. The type of pharmacopuncture solution used in the intervention, the total dose administered (mL), and the depth of needle insertion were recorded.

### 2.6. Outcomes Measures

Outcomes assessments were administered by Korean medicine doctors (KMDs) with no less than 3 years of clinical experience who acquired their KMD license upon completing the 6-year course at the College of Korean Medicine. The outcome assessor did not participate in the intervention and conducted the assessment in a separate space to ensure blinding. At the screening stage, information on patient characteristics, such as sex, age, weight, smoking history, history of alcohol consumption, medical history, and present illness, was collected. Baseline measurements were made on hospitalization day 2 before the intervention, and the primary endpoint was assessed using the measurements on day 4 after the intervention, immediately after completion of three sessions of pharmacopuncture on the suboccipital muscles.

#### 2.6.1. Primary Outcome

In this study, the primary outcome was the difference in the change in headache NRS scores between the two groups, measured after three sessions of suboccipital muscle pharmacopuncture. The NRS is a numeric pain scale for the objective representation of the subjective pain felt by a patient. Patients were asked to rate the severity of pain using scores ranging from 0 to 10, with 0 indicating no pain and 10 indicating the worst pain imaginable. NRS scores for headache were measured at screening, baseline, before and after treatment (from one to three times depending on the individual patient), after treatment on hospitalization day 4, the day of discharge, and 2 months after enrollment.

#### 2.6.2. Secondary Outcomes

In this study, the secondary outcomes were changes in the NRS scores for neck pain, Headache Disability Index (HDI), Headache Impact Test (HIT-6), EuroQol 5-Dimension (EQ-5D) related to the evaluation of quality of life, and Patient Global Impression of Change (PGIC).

(1) NRS for neck pain

Each patient was asked to rate the severity of pain by selecting a number from among the scores ranging from 0 to 10, with zero indicating no pain and 10 indicating the worst unbearable pain. NRS scores for neck pain were measured at baseline, before and after treatment (from one to three times depending on the individual patient), after treatment on hospitalization day 4, the day of discharge, and 2 months after enrollment.

(2) Headache Disability Index (HDI)

The HDI is a useful scale developed for the quantitative evaluation of the impact of headaches, their treatment on daily living, and the impact of headaches on daily living. It is a 25-item questionnaire subgrouped into functional and emotional subscales. The score ranged from 0 to 100, with higher scores indicating more severe headaches. HDI scores were measured at baseline, after treatment on hospitalization day 4, the day of discharge, and 2 months after enrollment [24].

(3) Headache impact test-6 (HIT-6)

The HIT-6 was developed to assess the impact of various types of headaches, including tension-type headaches and migraines. It is a self-report form covering 4 weeks with six questions for assessing pain, social functioning, role functioning, cognitive functioning, psychological distress, and vitality. In HIT-6, the scores for the impact of headache are categorized as follows: ≤49 for no or little impact, 50–55 for some impact, 56–60 for substantial impact, and > 60 for severe impact. HIT-6 scores were measured at baseline, after treatment on hospitalization day 4, the day of discharge, and 2 months after enrollment [25].

(4) Quality of life: EuroQol 5-Dimension (EQ-5D)

The EuroQol 5-dimension (EQ-5D) is an instrument developed to assess health-related quality of life and is widely used in the healthcare sector. The EQ-5D-5L consists of five items of multiple-choice questions assessing the participant’s current health state (mobility, self-care, usual activities, pain/discomfort, and anxiety/depression), and five response levels are used for assessment. In this study, the weights for health-related quality of life were calculated by applying a weight model that provides estimates for the Korean population. The EQ-5D questionnaire was administered at baseline, after treatment on hospitalization day 4, the day of discharge, and 2 months after enrollment [26].

(5) Patient Global Impression of Change (PGIC)

PGIC is a scale for self-assessment of the degree of improvement. Participants subjectively rated their changes on a 7-point Likert scale. The PGIC was measured after treatment on hospitalization day 4, the day of discharge, and 2 months after enrollment [27].

### 2.7. Sample Size Calculation

Regarding sample size, there were no previous studies that could be used as reference in calculating the sample size for the study. A previous study reported that 26–34 participants would be needed per group to obtain statistically significant results from a randomized controlled trial [28]. Comprehensively considering the above study results, recruitment possibilities, and the institution’s budget and human resources, we decided to recruit 40 patients in each group, a total of 80 patients, through an internal meeting of the research team.

### 2.8. Adverse Events

Adverse events (AEs) refer to any unfavorable and unintended signs, symptoms, or diseases that occur after an intervention in a clinical trial. However, not all events have a causal relationship with the intervention. In this study, all AEs, starting from admission during the study participation period, were investigated regardless of the causal relationship status with the study interventions. Information on AEs was collected through the self-reported symptoms by the patients and the observations by the researchers.

The severity of AEs was classified into three levels according to the classification by Spilker et al., as follows: (1) mild—symptoms requiring no additional treatment with no functional disruption to the participant’s normal activities of daily living (ADLs); (2) moderate—symptoms causing a significant functional disruption to the participant’s normal ADLs, which may require treatment and disappear over time when additional treatment is applied; and (3) severe—symptoms requiring immediate advanced treatment because of severity of the symptoms, resulting in sequelae [29]. For the assessment of causality between the study interventions and the AEs, a scale with six levels presented by the World Health Organization-Uppsala Monitoring Center causality assessment system was used (1 = related, 2 = probably related, 3 = possibly related, 4 = probably not related, 5 = not related, and 6 = unknown) [30].

### 2.9. Statistical Analysis

This study conducted intention-to-treat (ITT) and per-protocol (PP) analyses, with the ITT as the primary analysis. Participants who underwent at least two treatment sessions were analyzed separately for PP analysis.

The sociodemographic characteristics of the participants were evaluated for each group. Continuous variables were expressed as the mean (standard deviation) or the median (quartile), and differences between the groups were analyzed using the Independent Samples *t*-test. For categorical variables, the chi-square test was used to analyze between-group differences.

The efficacy endpoint of this clinical study was the difference in the changes in continuous outcomes at each time point from the baseline values between the two groups. A linear mixed model (LMM) was implemented for the primary analysis, with the baseline value of each variable as the covariate and the assigned group as the fixed factor. The difference in change from baseline at each time point in the two groups was presented along with the 95% CI and *p* value, and Cohen’s d value was also presented. Meanwhile, since PGIC does not have a baseline value, it represents the difference in values at each time point rather than the difference in change from the baseline.

Missing values were handled with the mixed models for repeated measures. For sensitivity analysis, missing values were handled using multiple imputations, and the last observation carried forward (LOCF) methods were used to analyze variance. In addition, the between-group difference in the total follow-up time and the area under the curve (AUC) between the baseline and the primary endpoint assessment were calculated using the trapezoidal rule.

Furthermore, survival analysis was performed by defining a decrease in pain by more than half as an event. Survival analysis was performed using the NRS score for headache, NRS for neck pain, and HDI. All statistical analyses were performed using the statistical package SAS (version 9.1.3; SAS Institute, Inc., Cary, NC, USA), and the statistical significance level was set at *p* < 0.05.

## 3. Results

### 3.1. Participants and Their Baseline Characteristics

**Figure 2:**
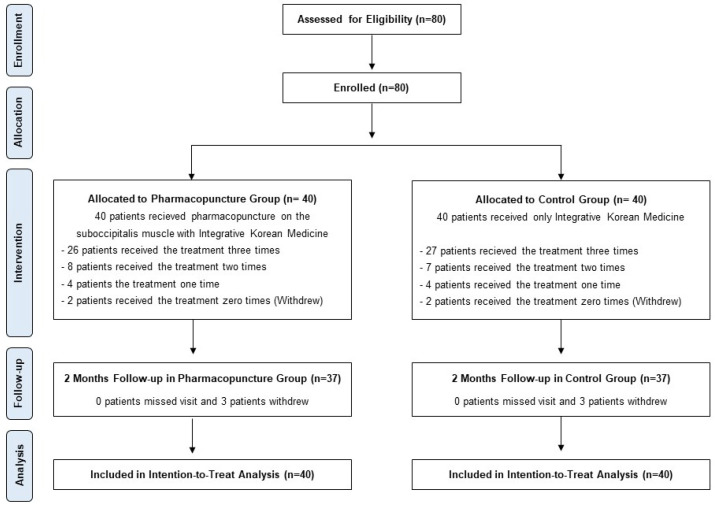
Participant flow diagram.

Between October 2022 and January 2023, inpatients who complained of TA-induced acute tension headaches were screened, and 80 patients who met the inclusion criteria were enrolled in this study. Forty patients were randomized into the suboccipital muscle pharmacopuncture group (pharmacopuncture group) or the IKM treatment group (comparison group). Six patients dropped out: three from the pharmacopuncture group and three from the comparison group. The reason for dropouts in the pharmacopuncture group was that one participant withdrew their consent to participate at a 2-month follow-up, and the other two participants were discharged on day 2 immediately after their enrollment. In the comparison group, two participants were discharged immediately after their enrollment on day 2, and one participant dropped out after patient enrollment and the first treatment session because of a history of medication for mental illness that may affect the outcomes of the study. In both the pharmacopuncture and comparison groups, 37 patients completed all treatment sessions and participated in the follow-up after 2 months. ITT analysis was conducted on 40 participants in each group (Figure 2).

As for the gender distribution of the participants, men accounted for 57.6% and 60% of the pharmacopuncture and comparison groups, respectively, indicating a slightly higher proportion of men than women in both groups. The mean age of the participants was 43.10 ± 14.05 in the pharmacopuncture group and 41.55 ± 15.74 in the comparison group. In the pharmacopuncture group, the mean NRS score at admission was 6.97 ± 0.28 and 6.85 ± 0.49 in the comparison group. The results confirmed no significant differences in baseline characteristics between the two groups (Table 1).

### 3.2. Treatment

Regarding the types of pharmacopuncture solution used in the interventions, Shinbaro Pharmacopuncture was most frequently used by 15 participants (37.5%), followed by Hwangryunhaedok-tang Pharmacopuncture by nine participants (22.5%), muscle relaxation pharmacopuncture by nine participants (22.5%), and Jungsongouhyul Pharmacopuncture by seven participants (17.5%). For each session of pharmacopuncture, a mean volume of 1.87 ± 1.15 mL of the solution was used, with the minimum volume being 0.5 mL for 15 participants (30%) and the maximum volume being 4 mL for one participant (2.5%). The mean depth of needle insertion was 7.70 ± 1.95 mm, with a minimum depth of 5 mm in two participants (5.0%) and a maximum depth of 11 mm in three participants (7.5%) (Supplementary Table S2).

### 3.3. Outcome Comparison between the Two Groups

**Figure 3:**
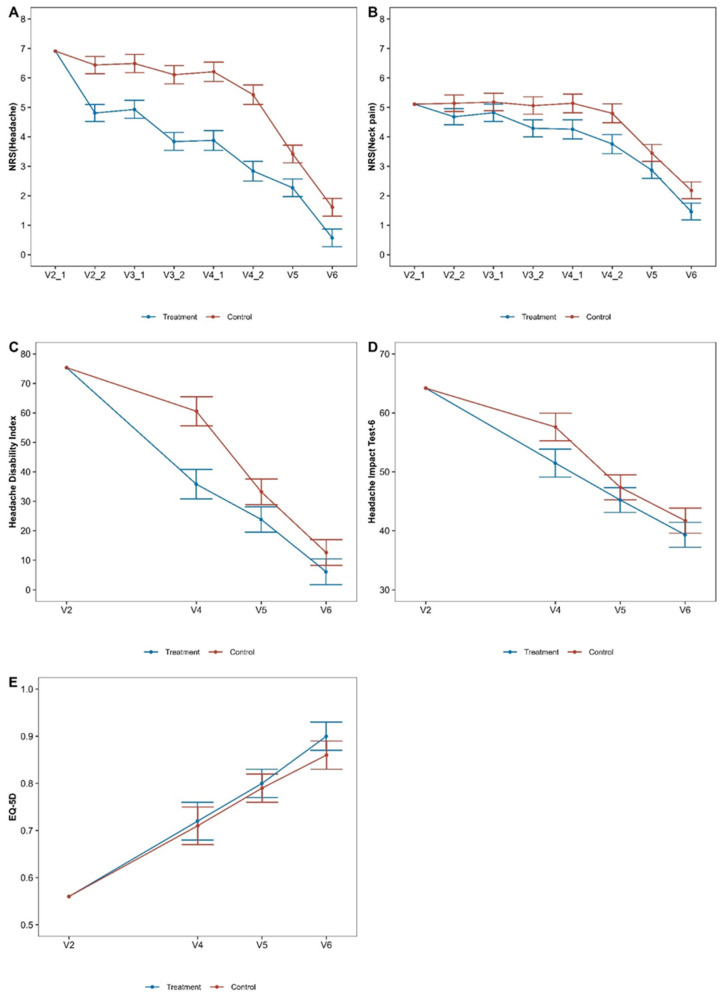
Comparison of the outcomes at each measuring point between the two groups (ITT). (**A**) Numeric rating scale for headache, (**B**) Numeric rating scale for neck pain, (**C**) Headache disability index, (**D**) Headache Impact Test-5, (**E**) EuroQol 5-Dimension.

An LMM of the mixed model for repeated measures was used to compare outcomes between the two groups, and the analysis results are presented and illustrated in Table 2 and Figure 3, respectively. Assessment of the primary endpoint, which was the difference between the two groups in the NRS score for headache measured after treatment on day 4, and the other outcomes, such as the NRS scores for neck pain, HDI, and HIT-6, confirmed a significantly superior improvement of symptoms in the group with pharmacopuncture on the suboccipital muscles as add-on therapy (pharmacopuncture group) than in the group with IKM treatment alone (comparison group) (difference in NRS of headache −2.59, 95% CI −3.06 to −2.12; NRS of Neck pain −1.05, 95% CI, −1.50 to −0.59; HDI −24.78, 95% CI, −31.79 to −17.76; HIT-6 −6.13, 95% CI, −9.47 to −2.78).

Furthermore, at the day of discharge(difference in NRS of headache −1.15, 95% CI, −1.57 to −0.73; NRS of neck pain −0.58, 95% CI, −0.98 to −0.18; HDI −9.37, 95% CI, −15.48 to −3.26) and at follow-up after 2 months(difference in NRS of headache −1.03, 95% CI, −1.46 to −0.61; NRS of neck pain −0.72, 95% CI, −1.12 to −0.32; HDI −6.51, 95% CI, −12.65 to −0.37) from enrollment, statistically significant and superior effectiveness was confirmed in the pharmacopuncture group than in the comparison group in terms of the NRS score for headache, NRS for neck pain, and HDI.

Similar trends were observed in the results of PP analysis, LMM ITT analysis, and sensitivity analysis using LOCF and MI, confirming significantly superior improvements in the pharmacopuncture group than in the comparison group in the assessment of NRS for headache, NRS for neck pain, and HDI (Supplementary Tables S3–S5). Regardless of the analysis method, for the NRS score for headache, which is the primary outcome, the degree of improvement in the pharmacopuncture group was significantly greater than that in the comparison group at the time of primary endpoint assessment, on the day of discharge, and follow-up.

As a result of analyzing the cumulative values of each outcome at the follow-up timepoint of 2 months using the AUC calculation, the pharmacopuncture group showed statistically significantly more effective outcomes in terms of the assessment of NRS for headache, NRS for neck pain, and HDI (AUC difference in NRS score for headache, −62.47, 95% CI, −88.80 to −36.15; NRS score for neck pain, −33.76, 95% CI, −61.76 to −5.77; HDI, −470.48, 95% CI, −773.79 to −167.17) (Table 3).

### 3.4. Survival Analysis

**Figure 4:**
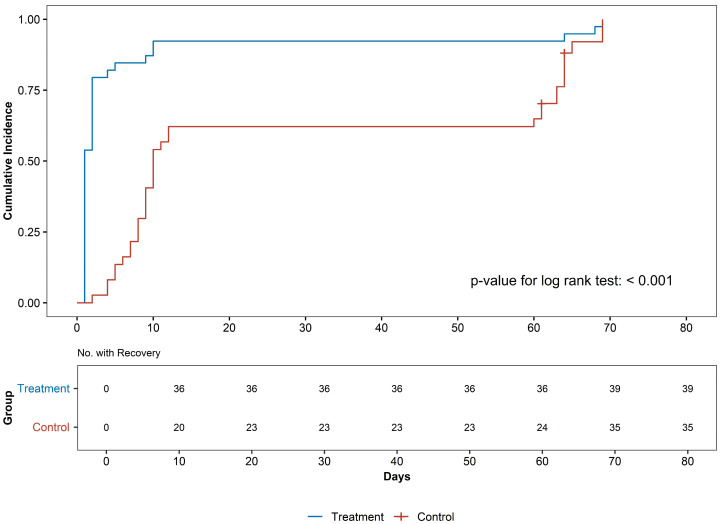
Kaplan–Meier curves for the probability of recovery in the treatment and comparison groups.

Recovery was defined as a decrease in the NRS score for headache and neck pain by more than half [17,31]. The survival analysis of recovery was performed based on the definition of y. The average recovery in the pharmacopuncture group for NRS score for headache was 1 (95% CI: 1–2), whereas in the comparison group, it was 9 (95% CI: 9–61). The NRS score for neck pain was 11 (95% CI: 9–65) and 69 (95% CI: 68–72), respectively. The pharmacopuncture group showed a faster recovery rate for both outcome measures than the comparison group (*p* < 0.001 by low-rank test) (Figure 4).

### 3.5. Adverse Events

Fourteen adverse events were reported, with six cases in the integrated Korean medicine treatment group and six in the pharmacopuncture group. A total of one case of AE that was considered likely to have a causal relationship with the study occurred in the pharmacopuncture group, while the comparison group had zero cases. One participant in the pharmacopuncture group complained of redness at the intervention site after the third pharmacopuncture session; however, the symptoms were alleviated after a week without additional treatment. AEs without a causal relationship with the study intervention were as follows: four cases of muscle cramps, four cases of sore throat, three cases of dyspepsia and nausea, and two cases of insomnia/sleep disturbance. No serious adverse events (SAEs) were recorded (Table 4).

## 4. Discussion

In this study, we compared the outcomes of IKM treatment alone and pharmacopuncture on the suboccipital muscles as an add-on therapy to IKM treatment for patients complaining of acute tension headaches among the symptoms of TA-induced WADs. Both groups improved in reducing headache and neck pain and enhancing the quality of life. However, the improvement was greater and faster in the case of combination with pharmacopuncture on the suboccipital muscles than in the case of IKM treatment alone. Furthermore, at follow-up after 2 months, the pharmacopuncture group showed a higher quality of life and treatment satisfaction than the comparison group. No SAEs occurred in either group, confirming the safety of the treatment methods.

Meanwhile, a pragmatic clinical trial (PCT) evaluates the overall effectiveness of treatment as clinical research aimed at providing information on decision-making in clinical practice [32]. PCT effectively reflects daily clinical treatment content, aligning with real medical settings, thus adhering to treatment and management methods consistent with routine clinical practice. In this study, a pragmatic study design was adopted to enhance external validity, aiming to assess the efficacy of a specific pharmacopuncture solution and evaluate the effectiveness of pharmacopuncture treatment [33]. The selection of interventions, including the pharmacopuncture type, dosage, depth of needling for suboccipital muscles, and the type and location of integrated traditional Korean medicine treatments, was left to physicians’ clinical judgment [34].

In the pharmacopuncture group, the reduction in the NRS score for headache, HDI, and NRS for neck pain on hospitalization day 4, the day of discharge, and at follow-up after 2 months was greater than that in the comparison group. In addition, it is generally known that the Minimal Clinically Important Difference (MCID) for the NRS, HDI, and HIT-6 are 2, 29, and 8, respectively [24,35,36,37]. In the case of the pharmacopuncture group, at hospitalization day 4, which is the time point for assessing the primary endpoint, the changes in the NRS score for headache, HDI, and HIT-6 were greater than the reported MCID values. However, in the comparison group, outcome changes in the primary endpoint were smaller than the MCID values, indicating insufficient treatment efficacy. In both groups, on the day of discharge and at follow-up after 2 months, the headache outcomes, which are NRS for headache, HDI, and HIT-6, decreased by more than the MCID.

Meanwhile, one of the outcome measures, HIT-6, showed a trend different from that of other pain outcomes, such as HDI. There was a significant difference in the change in HIT-6 scores between the two groups at the time of the primary endpoint assessment (difference; −6.13, 95% CI −9.47 to −2.78, *p* < 0.001). However, there was no significant difference between the two groups on the day of discharge (difference; −2.15, 95% CI −5.12 to 0.81, *p* = 0.154) or at follow-up after 2 months (difference; −2.41, 95% CI −5.39 to 0.57, *p* = 0.111). The difference between the HDI and HIT-6 is that the HDI is used to assess the functional and emotional impacts of headache on daily living at the time of assessment, whereas HIT-6 is used to assess social functioning, role functioning, vitality, cognitive functioning, and psychological distress over the last 4 weeks [38,39]. The interpretation of this difference is that the symptoms in both the pharmacopuncture and comparison groups were significantly improved compared to those at the baseline. As a result, the impact of headaches on daily living was reduced. Consequently, the HIT-6, which assesses social functioning, role functioning, etc., may no longer show significant differences between the two groups. Another point worth noting is that there was no significant difference between the two groups in terms of quality of life and treatment satisfaction on hospitalization day 4 and the day of discharge; however, at follow-up after 2 months, both quality of life and treatment satisfaction were significantly higher in the pharmacopuncture group than in the comparison group.

Sung et al. reported the presence of multiple active TrPs in the suboccipital area as a characteristic of patients with chronic tension-type headaches (CTTH), distinguishing them from patients with regular headaches [40]. Palacios-Ceña et al. demonstrated both CTTH and frequent episodic tension-type headache patients exhibited a positive correlation between the number of active TrPs and the severity and frequency of headache as well as trait anxiety [41]. In addition, Matteo et al. reported that patients with WADs had more active TrPs than other patients with mechanical neck pain and that pain intensity increased with the number of active TrPs, emphasizing the importance of TrP treatment in the treatment strategy for patients with WADs [42]. In careful consideration of the above previous studies, it is thought that the reason for the pharmacopuncture group showing high quality of life and satisfaction at follow-up after 2 months is that pharmacopuncture on the suboccipital muscles worked for the treatment of active TrPs in the suboccipital muscles, effectively relieving pain and preventing symptoms from progressing to chronic conditions.

The main area of treatment in this study is the suboccipital muscles, which have been reported to maintain head stability while allowing delicate comparison of the movement of the atlanto-occipital and atlanto-axial joints with a weak, sustained force [9]. Previous studies have reported an association between suboccipital muscles and headache and neck pain [9,10,11,12,13]. Although the exact pathogenesis remains to be elucidated, myofascial TrPs and muscle tenderness developed by muscle injuries contribute to the sensitization of peripheral nociceptors, causing central sensitization and increasing the input of noxious stimuli, which causes pain [9,10,12,40].

Thus, studies have been conducted with different methods for treating problems in the suboccipital muscles, including manual therapy [10], physical therapy [40], deep dry needling [11], and posture correction exercises [40]. These previous studies reported the outcomes of reduced headache and neck pain and improvements in physical function. In particular, Gemma et al. reported that when the combined treatment of physical therapy and manual therapy was administered for 4 weeks, improved outcomes were obtained, with the HIT-6 and HDI reduced from 60.75 to 56.70 and 21.67 to 20.95, respectively [10]. Additionally, Cho et al. reported that when the combination of physical therapy and forehead position correction exercise was administered for 4 weeks, HIT-6 scores decreased from 59.25 ±5.08 to 44.58 ±8.32 [40]. Although direct comparisons between the results of this study and those of previous studies may not be possible, in this study, after 3 days of pharmacopuncture therapy sessions, the HDI and HIT-6 decreased from 75.35 to 35.78 and 64.20 to 51.47, respectively, indicating that pharmacopuncture has a marked therapeutic effect in a relatively short duration of treatment.

The treatment effects of pharmacopuncture are based on the interactions between the physical stimulation of acupuncture points (e.g., Ashi points, meridians, lesion sites or positive reaction points, and TrPs) and chemical stimulation from the active ingredients of herbal medicine [16,17]. The effects of physical stimulation of pharmacopuncture include the analgesic, muscle-relaxing, anti-inflammatory, and mild anxiolytic effects of acupuncture, which have been well documented [43], as well as the effects of irrigation and hydrodissection caused by the injection of the pharmacopuncture solution [16]. The chemical effects of pharmacopuncture vary depending on the active ingredients of the medicinal herbs used [44]. In particular, Shinbaro pharmacopuncture, the most frequently used type in this study, has been reported to modulate acute and chronic inflammatory processes and have neuroprotective and nerve regeneration-promoting effects [45]. These effects of Shinbaro pharmacopuncture contribute to restoring injured nerves, ligaments, and muscles and strengthening weakened tissues. Additionally, this pharmacopuncture is frequently used in musculoskeletal disorders [46]. Hyun et al. and Chang et al. reported that a combination of pharmacopuncture was more effective than acupuncture alone for treating TA-induced whiplash injury/neck pain [47,48]. Jang et al. reported a significant improvement in headaches after patients received pharmacopuncture [49]. However, these previous studies have limitations, such as the lack of randomization or small sample sizes.

Regarding the safety of the interventions, 14 adverse events (AEs) were reported among 12 patients in this study, all of which were mild. Regarding AEs that may have a causal relationship with the intervention, one case of redness at the site of intervention occurred in the pharmacopuncture group, and there were no AEs with causality in the comparison group. All 14 AEs in this study, including a single case of redness at the intervention site in the pharmacopuncture group, disappeared spontaneously without needing specific medical treatment.

This study has a few limitations. First, blinding the KMDs who performed the intervention and the patients was impossible because of the nature of the intervention. To minimize the bias caused by limitations in blinding, assessments were performed by medical staff members who were not involved in the intervention and were blinded to the group allocation. Second, because the comparison group underwent IKM treatment consisting of acupuncture, pharmacopuncture, and Chuna therapy, the participants may not have been greatly affected by the placebo effect during the study. Third, observation and monitoring of long-term prognosis were not possible since the outcomes at 2 months after enrollment were not collected. Future studies should investigate the long-term effects of the intervention by extending the follow-up period. Fourth, pharmacopuncture on the suboccipital muscles was not performed as a stand-alone intervention but as an add-on therapy in combination with other Korean medicine treatments. Therefore, to obtain high-quality evidence of the effectiveness of pharmacopuncture on the suboccipital muscles for TA-induced acute tension headache, further studies examining the effectiveness of pharmacopuncture on the suboccipital muscles as a single intervention are required. Furthermore, it would have been better if liver and kidney function had been followed up through blood tests. Lastly, caution is needed when interpreting the results because the sample size estimation was approximate.

Despite these limitations, this study has made significant contributions as the first pragmatic clinical trial to confirm that pharmacopuncture can be an effective and safe treatment for patients with TA-induced acute tension headaches. In addition, it was confirmed that IKM treatment could also be an effective treatment, and the effect was greater when combined with suboccipital muscle pharmacopuncture treatment.

In conclusion, pharmacopuncture on the suboccipital muscles could be an effective and safe treatment for patients with TA-induced acute tension headaches. This provides valuable insights into a novel and useful treatment method that can be applied in clinical practice in Korean medicine.

## Figures and Tables

**Table 1:** Baseline characteristics between the two groups.

	Total	Pharmacopuncture Group	Comparison Group	*p* Value
	N = 80	N = 40	N = 40	
Age				
The mean ± SD	42.33 ± 14.85	43.10 ± 14.05	41.55 ± 15.74	0.644
<30	19 (23.8)	8 (20.0)	11 (27.5)	0.613
30–39	17 (21.2)	7 (17.5)	10 (25.0)	
40–49	15 (18.8)	9 (22.5)	6 (15.0)	
50–59	16 (20.0)	10 (25.0)	6 (15.0)	
≥60	13 (16.2)	6 (15.0)	7 (17.5)	
Sex				
Male	47 (58.8)	23 (57.5)	24 (60.0)	1.000
Female	33 (41.2)	17 (42.5)	16 (40.0)	
Height (cm, the mean ± SD)	168.18 ± 8.21	168.05 ± 8.15	168.30 ± 8.37	0.893
Weight (kg, the mean ± SD)	69.01 ± 13.85	69.80 ± 15.40	68.22 ± 12.24	0.614
BMI				
the mean ± SD	24.30 ± 3.97	24.61 ± 4.58	23.99 ± 3.28	0.489
<25	46 (57.5)	23 (57.5)	23 (57.5)	1.000
≥25	34 (42.5)	17 (42.5)	17 (42.5)	
Alcohol consumption				
Yes	19 (23.8)	12 (30.0)	7 (17.5)	0.293
No	61 (76.2)	28 (70.0)	33 (82.5)	
Smoking				
Never-smoker	58 (72.5)	27 (67.5)	31 (77.5)	0.453
Former smoker	-	-	-	
Current smoker	22 (27.5)	13 (32.5)	9 (22.5)	
Occupation type				
Managers	-	-	-	0.395
Professionals and related occupations	7 (8.8)	4 (10.0)	3 (7.5)	
Office workers (clerical)	19 (23.8)	7 (17.5)	12 (30.0)	
Service and sales workers	13 (16.2)	8 (20.0)	5 (12.5)	
Skilled agricultural, forestry, and fishery workers	-	-	-	
Craft and related trades	3 (3.8)	3 (7.5)	-	
Plan and machine operators and assemblers	-	-	-	
Elementary occupations	11 (13.8)	6 (15.0)	5 (12.5)	
Armed forces	-	-	-	
No occupation (Including housewives and students)	27 (33.8)	12 (30.0)	15 (37.5)	
Onset type				
In car TA	74 (92.5)	36 (90.0)	38 (95.0)	0.675
out car TA	6 (7.5)	4 (10.0)	2 (5.0)	
Disease history				
Yes	35 (43.8)	17 (42.5)	18 (45.0)	1
No	45 (56.2)	23 (57.5)	22 (55.0)	
Baseline outcomes (the mean ± SD)				
Baseline NRS (Headache) (N = 79)	6.91 ± 0.40	6.97 ± 0.28	6.85 ± 0.49	0.156
Baseline NRS (Neck pain) (N = 79)	5.11 ± 0.39	5.12 ± 0.40	5.10 ± 0.38	0.801
Baseline HDI	61.81 ± 4.83	61.98 ± 4.89	61.65 ± 4.82	0.766
Baseline HIT-6	64.20 ± 3.07	64.05 ± 2.75	64.35 ± 3.40	0.665
Baseline EQ-5D	0.56 ± 0.13	0.54 ± 0.12	0.58 ± 0.13	0.110

BMI, basal metabolic rate; NRS, the numeric rating scale; HDI, headache Disability Index; HIT-6, Headache Impact Test-6; EQ-5D, EuroQol 5-Dimension; PGIC, Patient Global Impression of Change.

**Table 2:** Comparison of outcomes at each measuring point between the two groups (ITT).

		Baseline Hospitalization Day 2 (Before)	Hospitalization Day 2 (After)	Hospitalization Day 3 (Before)	Hospitalization Day 3 (After)	Hospitalization Day 4 (Before)	Hospitalization Day 4 (After)	Discharge	2 Months ±10 Days after Enrollment
		Visit 2 (Before)	Visit 2 (After)	Visit 3 (Before)	Visit 3 (After)	Visit 4 (Before)	Visit 4 (After)	Visit 5	Visit 6
NRS (Headache)	Pharmacopuncture group	6.91 (6.82 to 7.00)	4.81 (4.52 to 5.10)	4.93 (4.63 to 5.24)	3.84 (3.54 to 4.15)	3.88 (3.54 to 4.21)	2.84 (2.50 to 3.17)	2.27 (1.97 to 2.57)	0.57 (0.27 to 0.87)
	Comparison group		6.44 (6.14 to 6.73)	6.49 (6.18 to 6.80)	6.11 (5.80 to 6.42)	6.21 (5.88 to 6.54)	5.43 (5.10 to 5.76)	3.42 (3.12 to 3.72)	1.61 (1.31 to 1.91)
	Difference	—	−1.62 (−2.04 to −1.21)	−1.56 (−1.99 to −1.12)	−2.26 (−2.70 to −1.83)	−2.33 (−2.80 to −1.86)	−2.59 (−3.06 to −2.12)	−1.15 (−1.57 to −0.73)	−1.03 (−1.46 to −0.61)
	*p* value	—	<0.001 ***	<0.001 ***	<0.001 ***	<0.001 ***	<0.001 ***	<0.001 ***	<0.001 ***
	Cohen’s d	—	1.15	1.15	1.39	1.48	1.94	3.16	4.68
NRS (Neck pain)	Pharmacopuncture group	5.11 (5.03 to 5.20)	4.68 (4.41 to 4.96)	4.82 (4.52 to 5.11)	4.29 (4.00 to 4.58)	4.26 (3.93 to 4.58)	3.76 (3.43 to 4.08)	2.87 (2.59 to 3.16)	1.46 (1.18 to 1.75)
	Comparison group		5.14 (4.86 to 5.42)	5.18 (4.89 to 5.48)	5.06 (4.77 to 5.36)	5.14 (4.82 to 5.45)	4.80 (4.48 to 5.12)	3.45 (3.17 to 3.74)	2.18 (1.90 to 2.47)
	Difference	—	−0.46 (−0.85 to −0.07)	−0.37 (−0.78 to 0.05)	−0.78 (−1.19 to −0.36)	−0.88 (−1.33 to −0.43)	−1.05 (−1.50 to −0.59)	−0.58 (−0.98 to −0.18)	−0.72 (−1.12 to −0.32)
	*p* value	—	0.022 *	0.083	<0.001 ***	<0.001 ***	<0.001 ***	0.005 **	<0.001 ***
	Cohen’s d	—	0.38	0.43	0.69	0.68	0.97	1.75	2.18
HDI	Pharmacopuncture group	75.35 (73.75 to 76.95)					35.78 (30.77 to 40.79)	23.82 (19.51 to 28.13)	6.09 (1.73 to 10.44)
	Comparison group						60.56 (55.62 to 65.49)	33.19 (28.83 to 37.55)	12.59 (8.23 to 16.96)
	Difference	—					−24.78 (−31.79 to −17.76)	−9.37 (−15.48 to −3.26)	−6.51 (−12.65 to −0.37)
	*p* value	—					<0.001 ***	0.003 **	0.038 *
	Cohen’s d	—					1.59	2.66	5.65
HIT-6	Pharmacopuncture group	64.20 (63.53 to 64.87)					51.47 (49.09 to 53.86)	45.21 (43.11 to 47.30)	39.30 (37.19 to 41.41)
	Comparison group						57.60 (55.25 to 59.95)	47.36 (45.24 to 49.48)	41.71 (39.59 to 43.83)
	Difference	—					−6.13 (−9.47 to −2.78)	−2.15 (−5.12 to 0.81)	−2.41 (−5.39 to 0.57)
	*p* value	—					<0.001 ***	0.154	0.111
	Cohen’s d	—					1.52	2.30	4.51
EQ−5D	Pharmacopuncture group	0.56 (0.53 to 0.59)					0.72 (0.68 to 0.76)	0.80 (0.77 to 0.83)	0.90 (0.87 to 0.93)
	Comparison group						0.71 (0.67 to 0.75)	0.79 (0.76 to 0.82)	0.86 (0.83 to 0.89)
	Difference	—					0.01 (−0.04 to 0.06)	0.01 (−0.03 to 0.06)	0.05 (0.00 to 0.09)
	*p* value	—					0.688	0.582	0.047 *
	Cohen’s d	—					−1.56	−1.68	−2.05
PGIC	Pharmacopuncture group						3.22 (2.92 to 3.52)	2.34 (2.09 to 2.60)	2.06 (1.80 to 2.32)
	Comparison group						3.40 (3.10 to 3.70)	2.33 (2.07 to 2.58)	2.62 (2.36 to 2.88)
	Difference	—					0.18 (−0.25 to 0.61)	−0.02 (−0.38 to 0.35)	0.57 (0.20 to 0.93)
	*p* value	—					0.407	0.927	0.003 **
	Cohen’s d	—					−0.27	0.03	−0.66

NRS, the Numeric Rating Scale; HDI, headache Disability Index; HIT-6, Headache Impact Test-6; EQ-5D, EuroQol 5-Dimension; PGIC, Patient Global Impression of Change. *p* values are indicated alongside the estimated differences as follows: * *p* < 0.05; ** *p* < 0.01; *** *p* < 0.001.

**Table 3:** Area under the curve of outcomes according to treatment (ITT).

	Pharmacopuncture Group	Comparison Group	Difference (95% CI)	*p* Value	Cohen’s d
NRS (Headache)	100.76 (82.31 to 119.22)	163.24 (144.53 to 181.95)	−62.47 (−88.80 to −36.15)	<0.001 ***	1.10
NRS (Neck pain)	143.20 (123.39 to 163.01)	176.96 (156.87 to 197.06)	−33.76 (−61.76 to −5.77)	0.019 *	0.57
HDI	1029.29 (818.04 to 1240.53)	1499.77 (1282.76 to 1716.77)	−470.48 (−773.79 to −167.17)	0.003 **	0.74
HIT-6	2611.98 (2504.32 to 2719.64)	2743.06 (2633.25 to 2852.86)	−131.08 (−284.70 to 22.55)	0.093	0.42
EQ5D	51.63 (49.99 to 53.27)	50.13 (48.48 to 51.77)	1.50 (−0.82 to 3.82)	0.201	−0.17

NRS, the Numeric Rating Scale; HDI, headache Disability Index; HIT-6, Headache Impact Test-6; EQ-5D, EuroQol 5-Dimension; PGIC, Patient Global Impression of Change. *p* values are indicated alongside the estimated differences as follows: * *p* < 0.05; ** *p* < 0.01; *** *p* < 0.001.

**Table 4:** Cases of adverse events during the study period by treatment group.

	Pharmacopuncture Group (N = 40)	Comparison Group(N = 40)
Total cases of adverse events	7	7
Possibility of a causal relationship	1	0
Redness at the site of intervention	1	0
No possibility of a causal relationship	6	7
Muscle cramps	1	3
Sore throat	2	2
Dyspepsia and nausea	1	2
Insomnia/sleep disturbance	2	0
Serious adverse events	0	0

## Data Availability

The data supporting this study’s findings are available from the corresponding author upon reasonable request.

## Supplementary Materials

The following supporting information can be downloaded at: https://www.mdpi.com/article/10.3390/jcm13154457/s1, Table S1: Study schedule and measurements at each visit; Table S2: Details of pharmacopuncture treatment used in the pharmacoacupuncture group; Table S3: LMM (PP); Table S4: LOCF ANCOVA (ITT); Table S5: MI ANCOVA (ITT).
