# Supplementary material for: Effectiveness and Safety of Pharmacopuncture on Inpatients with Tension Headache Caused by Traffic Accidents: A Pragmatic Randomized Controlled Trial
Source: J Clin Med. 2024 Jul 30;13(15):4457. doi: 10.3390/jcm13154457 (PMC11312919; doi:10.3390/jcm13154457)
Supplement: Supplementary file 1 [file jcm-13-04457-s001.zip › jcm-3076955-supplementary.pdf]

Supplementary table S1. Study schedule and measurements at each visit

Time point	Study period					
	Screening	Active treatment				Follow up
Day	Hospitalization Day 1 (visit 1)	Hospitalization Day 2 (visit 2) Base line	Hospitalization Day 3 (v3)	Hospitalization Day 4 (v4) Primary end point	Discharge (v5)	**2 months ±10 days after enrollment** (v6)
Enrollment						
Informed consent form	○					
Sociodemographic characteristics	○					
Medical history (Headache, and other)	○					
Confirm suitability for study	○					
Randomized allocation		○				
Intervention						
Treatment in integrative Korean medicine treatment& Pharmacopuncture on suboccipital muscle group	△ (only integrative Korean medicine)	○	○	○	△ (only integrative Korean medicine)	
Treatment in integrative Korean medicine treatment group	○	○	○	○	○	
Assessment						
NRS of Headache	○	○*	○*	○*	○	○
NRS of Neck pain		○¶	○¶	○¶	○	○
HDI		○†		○♀	○	○
HIT-6		○†		○♀	○	○
EQ-5D		○†		○♀	○	○
PGIC				○♀	○	○
Adverse events		○	○	○	○	○

For baseline measurements, the outcome values measured on Day 2 of hospitalization before initiation of any treatment were used, and for primary endpoint measurements the outcome values measured on Day 4 of hospitalization after completing all treatment sessions were used If Day 4 of hospitalization falls on the same date as the discharge date, the outcome assessment values on Day 4 of hospitalization can be used as substitutes for outcomes assessment on the discharge date. The time window for follow-up 2 months after the enrollment date was set to be ±10 days. * Additional NRS of Headache after treatment. ¶ Additional NRS of Neck pain after treatment. † Assessed before treatment. ♀ Assessed after treatment

Supplementary Table S2. Details of pharmacopuncture treatment used in the pharmacoacupuncture group

Supplementary Table 2. Treatment Details				
Type of pharmacopuncture (N=39)				
Shinbaro	15 (37.5)	15 (37.5)	-	
Hwangryunhaedok	9 (22.5)	9 (22.5)	-	
Jungsongouhyul	7 (17.5)	7 (17.5)	-	-
Muscle Relaxation	9 (22.5)	9 (22.5)	-	
Hominis Placenta	-	-	-	
Purified Essential Bee Venom	-	-	-	
One-time dose (mL)				
Mean ± SD	1.87 ± 1.15	1.87 ± 1.15	-	-
0.5	12 (30.0)	12 (30.0)	-	
0.8	1 (2.5)	1 (2.5)	-	
1	4 (10.0)	4 (10.0)	-	
2	6 (15.0)	6 (15.0)	-	-
3	16 (40.0)	16 (40.0)	-	
4	1 (2.5)	1 (2.5)	-	
Depth of needle insertion (mm)				
Mean ± SD	7.70 ± 1.95	7.70 ± 1.95	-	-
5	2 (5.0)	2 (5.0)	-	
6	16 (40.0)	16 (40.0)	-	
7	3 (7.5)	3 (7.5)	-	
8	6 (15.0)	6 (15.0)	-	-
10	10 (25.0)	10 (25.0)	-	
11	3 (7.5)	3 (7.5)	-	

Supplementary Table S3. LMM (PP)

		Hospitalization Day 2(Before)	Hospitalization Day 4 (After)	Discharge	2 months ±10 days after enrollment
		Visit 2 (Before)	Visit 4 (After)	Visit 5	Visit 6
NRS (Headache)	Suboccipital muscles pharmacopuncture group(N=34)	6.93 (6.83 to 7.02)	2.77 (2.46 to 3.09)	2.16 (1.87 to 2.45)	0.35 (0.05 to 0.64)
	IKM group (N=34)		5.40 (5.09 to 5.72)	3.32 (3.02 to 3.61)	1.55 (1.26 to 1.84)
	Difference	—	-2.63 (-3.08 to -2.18)	-1.15 (-1.57 to -0.74)	-1.20 (-1.62 to -0.79)
	P value	—	<0.001***	<0.001***	<0.001***
NRS (Neck pain)	Suboccipital muscles pharmacopuncture group	5.13 (5.03 to 5.23)	3.69 (3.40 to 3.97)	2.72 (2.46 to 2.98)	1.23 (0.97 to 1.49)
	IKM group		4.76 (4.48 to 5.04)	3.32 (3.06 to 3.58)	2.12 (1.86 to 2.38)
	Difference	—	-1.07 (-1.48 to -0.67)	-0.60 (-0.97 to -0.23)	-0.89 (-1.26 to -0.52)
	P value	—	<0.001***	0.001**	<0.001***
HDI	Suboccipital muscles pharmacopuncture group	75.12 (73.39 to 76.85)	35.18 (30.17 to 40.19)	22.82 (18.33 to 27.31)	5.24 (0.69 to 9.78)
	IKM group		59.81 (54.88 to 64.74)	31.95 (27.45 to 36.46)	11.66 (7.15 to 16.17)
	Difference	—	-24.63 (-31.65 to -17.61)	-9.13 (-15.48 to -2.79)	-6.42 (-12.81 to -0.04)
	P value	—	<0.001***	0.005**	0.049*
HIT-6	Suboccipital muscles pharmacopuncture group	64.01 (63.29 to 64.74)	50.99 (48.65 to 53.32)	44.62 (42.51 to 46.73)	38.70 (36.57 to 40.83)
	IKM group		57.11 (54.81 to 59.40)	46.78 (44.67 to 48.90)	41.17 (39.05 to 43.28)
	Difference	—	-6.12 (-9.39 to -2.85)	-2.16 (-5.14 to 0.82)	-2.47 (-5.47 to 0.53)
	P value	—	<0.001***	0.154	0.105
EQ5D	Suboccipital muscles pharmacopuncture group	0.57 (0.54 to 0.60)	0.73 (0.71 to 0.76)	0.83 (0.81 to 0.85)	0.92 (0.90 to 0.94)
	IKM group		0.74 (0.71 to 0.76)	0.83 (0.81 to 0.85)	0.88 (0.86 to 0.90)
	Difference	—	0.00 (-0.04 to 0.03)	0.00 (-0.03 to 0.03)	0.04 (0.01 to 0.07)
	P value	—	0.938	0.910	0.013*
PGIC	Suboccipital muscles pharmacopuncture group		3.14 (2.86 to 3.42)	2.18 (1.93 to 2.42)	1.91 (1.66 to 2.16)
	IKM group		3.35 (3.08 to 3.62)	2.18 (1.93 to 2.42)	2.59 (2.34 to 2.83)
	Difference	—	0.21 (-0.18 to 0.60)	0.00 (-0.35 to 0.35)	0.68 (0.33 to 1.02)
	P value	—	0.294	1.000	<0.001***

Supplementary Table S4. LOCF ANCOVA (ITT)

		Hospitalization Day 4 (After)	Discharge	2 months ±10 days after enrollment
		Visit 4 (After)	Visit 5	Visit 6
NRS (Headache)	Suboccipital muscles pharmacopuncture group	2.71 (2.40 to 3.03)	2.36 (2.00 to 2.71)	0.62 (0.25 to 0.99)
	IKM group	5.27 (4.96 to 5.58)	3.42 (3.06 to 3.78)	1.60 (1.24 to 1.97)
	Difference	-2.56 (-3.00 to -2.11)	-1.07 (-1.57 to -0.56)	-0.98 (-1.50 to -0.47)
	P value	<0.001***	<0.001***	<0.001***
NRS (Neck pain)	Suboccipital muscles pharmacopuncture group	3.75 (3.22 to 4.27)	2.93 (2.59 to 3.26)	1.52 (1.08 to 1.97)
	IKM group	4.81 (4.25 to 5.36)	3.47 (3.12 to 3.81)	2.16 (1.71 to 2.60)
	Difference	-1.06 (-1.51 to -0.61)	-0.54 (-1.01 to -0.06)	-0.63 (-1.26 to -0.01)
	P value	<0.001***	0.027*	0.048*
HDI	Suboccipital muscles pharmacopuncture group	34.14 (28.63 to 39.65)	23.88 (18.62 to 29.13)	6.33 (3.45 to 9.22)
	IKM group	57.72 (52.35 to 63.10)	33.21 (27.89 to 38.52)	12.61 (9.73 to 15.49)
	Difference	-23.58 (-31.28 to -15.89)	-9.33 (-16.76 to -1.90)	-6.27 (-10.33 to -2.22)
	P value	<0.001***	0.015*	0.003**
HIT-6	Suboccipital muscles pharmacopuncture group	50.60 (48.14 to 53.06)	45.15 (42.61 to 47.70)	39.24 (37.65 to 40.83)
	IKM group	56.07 (53.66 to 58.47)	47.26 (44.69 to 49.84)	41.59 (40.00 to 43.18)
	Difference	-5.47 (-8.93 to -2.01)	-2.11 (-5.71 to 1.50)	-2.35 (-4.59 to -0.11)
	P value	0.003**	0.248	0.040*
EQ5D	Suboccipital muscles pharmacopuncture group	0.74 (0.72 to 0.77)	0.81 (0.77 to 0.84)	0.90 (0.87 to 0.94)
	IKM group	0.73 (0.71 to 0.75)	0.79 (0.75 to 0.83)	0.86 (0.83 to 0.90)
	Difference	0.01 (-0.02 to 0.05)	0.02 (-0.03 to 0.07)	0.04 (-0.01 to 0.08)
	P value	0.530	0.513	0.098

* Since the assessment of PGIC requires the assumption that the previous state/condition will be maintained, we considered its appropriateness for inclusion in this table and as a result, the PGIC values were not included because it was not the primary outcome.

Supplementary Table S5. MI ANCOVA (ITT)

		Hospitalization Day 4 (After)	Discharge	2 months ±10 days after enrollment
		Visit 4 (After)	Visit 5	Visit 6
NRS (Headache)	Suboccipital muscles pharmacopuncture group	2.87 (1.48 to 4.27)	2.37 (2.01 to 2.72)	0.67 (0.31 to 1.04)
	IKM group	4.68 (3.46 to 5.90)	3.42 (3.06 to 3.78)	1.61 (1.24 to 1.98)
	Difference	-1.81 (-2.51 to -1.10)	-1.06 (-1.56 to -0.55)	-0.94 (-1.46 to -0.41)
	P value	<0.001***	<0.001***	<0.001***
NRS (Neck pain)	Suboccipital muscles pharmacopuncture group	3.58 (2.89 to 4.28)	2.92 (2.58 to 3.26)	1.58 (1.15 to 2.02)
	IKM group	4.30 (3.56 to 5.04)	3.45 (3.11 to 3.80)	2.16 (1.71 to 2.60)
	Difference	-0.72 (-1.22 to -0.21)	-0.54 (-1.01 to -0.06)	-0.57 (-1.19 to 0.04)
	P value	0.006**	0.029*	0.067
HDI	Suboccipital muscles pharmacopuncture group	38.38 (27.06 to 49.70)	24.20 (18.87 to 29.53)	6.34 (3.57 to 9.11)
	IKM group	55.47 (46.10 to 64.84)	33.16 (27.69 to 38.63)	12.70 (9.91 to 15.50)
	Difference	-17.09 (-26.57 to -7.61)	-8.96 (-16.50 to -1.43)	-6.36 (-10.27 to -2.45)
	P value	<0.001***	0.020*	0.002**
HIT-6	Suboccipital muscles pharmacopuncture group	52.01 (48.57 to 55.45)	45.30 (42.75 to 47.85)	39.31 (37.76 to 40.86)
	IKM group	55.53 (52.30 to 58.77)	47.15 (44.55 to 49.76)	41.57 (39.99 to 43.15)
	Difference	-3.52 (-7.41 to 0.36)	-1.86 (-5.44 to 1.73)	-2.26 (-4.48 to -0.04)
	P value	0.075	0.305	0.046*
EQ5D	Suboccipital muscles pharmacopuncture group	0.74 (0.71 to 0.76)	0.81 (0.77 to 0.84)	0.90 (0.87 to 0.93)
	IKM group	0.74 (0.71 to 0.77)	0.79 (0.76 to 0.83)	0.87 (0.83 to 0.90)
	Difference	0.00 (-0.04 to 0.04)	0.02 (-0.03 to 0.07)	0.04 (-0.01 to 0.08)
	P value	0.985	0.549	0.129
PGIC	Suboccipital muscles pharmacopuncture group	2.99 (2.66 to 3.32)	2.35 (2.12 to 2.58)	2.05 (1.77 to 2.33)
	IKM group	3.22 (2.91 to 3.53)	2.33 (2.10 to 2.56)	2.61 (2.32 to 2.89)
	Difference	0.23 (-0.20 to 0.66)	-0.02 (-0.34 to 0.30)	0.56 (0.16 to 0.95)
	P value	0.286	0.894	0.007**
